# Neuroprotective Effects of the Sonic Hedgehog Signaling Pathway in Ischemic Injury through Promotion of Synaptic and Neuronal Health

**DOI:** 10.1155/2020/8815195

**Published:** 2020-08-01

**Authors:** Sen Yin, Xuemei Bai, Danqing Xin, Tingting Li, Xili Chu, Hongfei Ke, Min Han, Wenqiang Chen, Xingang Li, Zhen Wang

**Affiliations:** ^1^Qilu Hospital, Cheeloo College of Medicine, Shandong University, Jinan, Shandong, China; ^2^Department of Physiology, School of Basic Medical Sciences, Cheeloo College of Medicine, Shandong University, Jinan, Shandong 250012, China

## Abstract

Cerebral ischemia is a common cerebrovascular condition which often induces neuronal apoptosis, leading to brain damage. The sonic hedgehog (Shh) signaling pathway has been reported to be involved in ischemic stroke, but the underlying mechanisms have not been fully elucidated. In the present study, we demonstrated that expressions of Shh, Ptch, and Gli-1 were significantly downregulated at 24 h following oxygen-glucose deprivation (OGD) injury in neurons *in vitro*, effects which were associated with increasing numbers of apoptotic cells and reactive oxygen species generation. In addition, expressions of synaptic proteins (neuroligin and neurexin) were significantly downregulated at 8 h following OGD, also associated with concomitant neuronal apoptosis. Treatment with purmorphamine, a Shh agonist, increased Gli-1 in the nucleus of neurons and protected against OGD injury, whereas the Shh inhibitor, cyclopamine, produced the opposite effects. Activation of Shh signals promoted CREB and Akt phosphorylation; upregulated the expressions of BDNF, neuroligin, and neurexin; and decreased NF-*κ*B phosphorylation following OGD. Notably, this activation of Shh signals was accompanied by improved neurobehavioral responses along with attenuations in edema and apoptosis at 48 h postischemic insult in rats. Taken together, these results demonstrate that activation of the Shh signaling pathway played a neuroprotective role in response to ischemic exposure via promotion of synaptic and neuronal health.

## 1. Introduction

Ischemic strokes are one of the leading causes of long-lasting disability in humans [1]. Cell death quickly results in neurons starved of oxygen and nutrients, and the subsequent excitotoxicity, oxidative stress, neuroinflammation, and apoptosis produce loss of structural and functional integrity of the brain [2]. Accordingly, novel therapeutic strategies for the restoration of central nervous system (CNS) integrity and promotion of functional recovery for patients with cerebral ischemia are urgently needed.

The sonic hedgehog (Shh) signaling pathway plays an important role in neurogenesis and neural patterning during development of the CNS [3]. When Shh binds to its receptor, Patched (Ptch), it depresses the G protein-coupled receptor Smoothened (Smo), leading to the activation of glioma-associated oncogene homolog 1 (Gli-1). Activated Gli-1 meditates the expression of many target genes that regulate cell growth, survival, and differentiation of cells, including neurons [4]. The Shh signaling pathway has been reported to be involved in ischemic stroke [5], as Shh expression was found to be upregulated in neurons during ischemia/hypoxia [6]. Moreover, inhibition of the Shh pathway exacerbated ischemic damage in rats, effects which were correlated with a downregulation in the expressions of Gli1 and Ptch [7]. Purmorphamine (PUR), a small molecular agonist of the Shh coreceptor, Smo, exerted protective effects in the middle cerebral artery occlusion (MCAO) model [8]. PUR, which has also been shown to promote blood-brain barrier formation, plays a crucial role in activation of the endogenous anti-inflammatory system within the CNS [9], and recent findings have indicated that PUR protects cortical neurons and restores neurological deficits after ischemic stroke in rats [10]. It has been reported by us that PUR exerted neuroprotection against subarachnoid hemorrhage-induced injury in adult rats [11] and hypoxia-ischemia in neonatal mice [12].

Despite this relatively substantial survey of information on Shh, details regarding the role of Shh signaling in ischemic stroke have not been fully elucidated. As one attempts to rectify this deficit, in this report, we focused on expression levels and effects of the Shh signaling pathway following oxygen-glucose deprivation (OGD) injury. In this way, it will be possible to examine some of the underlying neuroprotective mechanisms of the Shh signaling pathway in ischemic injury.

## 2. Materials and Methods

### 2.1. Reagents and Antibodies

The antibodies used for Western blot included anti-p-Akt (9271S, Cell Signaling Technology, USA), anti-Akt (9272S, Cell Signaling Technology, USA), anti-Shh (20697-1-AP, Proteintech Group, USA), anti-Gli-1 (ab151796, Abcam, USA), anti-Patch (ab53715, Abcam, USA), anti-NF-*κ*B (10745-1-AP, Proteintech Group, USA), anti-p-NF-*κ*B (3033S, Cell Signaling Technology, USA), anti-p-CREB (9198S, Cell Signaling Technology, USA), anti-CREB (9197S, Cell Signaling Technology, USA), anti-BDNF (17465-1-AP, Proteintech Group, USA), anti-neuroligin (ab186279, Abcam, USA), anti-neurexin (ab222806, Abcam, USA), and anti-*β*-actin (TA-09, Zhongshan Golden Bridge Biotechnology, China). The antibody for immunofluorescence was anti-Gli-1 (ab151796, Abcam, USA). PUR and cyclopamine (Cyc, a smoothened inhibitor) were purchased from Selleck Chemicals (Houston, TX, USA). Thread embolus was purchased from Guangzhou Jialing Biotechnology Co., Ltd. (L3800, Guangzhou, China). Annexin V-FITC/PI Double Labeling Apoptosis Detection Kit was purchased from BestBio Science (Shanghai, China). Terminal deoxynucleotidyl transferase-mediated dUTP-biotin nick end labeling (TUNEL) kit was purchased from KeyGen Biotech (KGA7073, Beyotime, Shanghai, China).

### 2.2. Cell Culture, Treatments, and Oxygen-Glucose Deprivation (OGD)

#### 2.2.1. Primary Neurons

Primary cortical neurons of mice were cultured as previously described [13]. Briefly, PND1 mice were used to harvest cortical neurons which were then cultured in serum-free neurobasal medium with 2% B27 and 1% penicillin-streptomycin. Cells were cultured in poly-D-lysine-coated twelve-well plates for 7 days and then treated with PUR (20 *μ*M) with/without Cyc (10 *μ*M).

To mimic the ischemic condition *in vitro*, cells were subjected to OGD. Briefly, cells were cultured at 37°C under 95% nitrogen and 5% CO_2_ for 6 h in glucose-free DMEM (125 mM NaCl, 2.8 mM KCl, 1.5 mM MgCl_2_, 0.05 mM MgSO_4_, 2 mM CaCl_2_, 0.83 mM NaH_2_PO_4_, 24 mM NaHCO_3_, and 2 mM HEPES). After OGD, primary neurons were placed in the original neurobasal medium with PUR (20 *μ*M) with/without Cyc (10 *μ*M) and returned to the incubator under normoxic conditions for the times indicated. Control cells were maintained under normal conditions without OGD. Cell apoptosis was assessed by TUNEL staining according to the manufacturer's protocol.

#### 2.2.2. PC12 Cells

PC12 cells were cultured in DMEM containing 5% FBS and 1% penicillin/streptomycin. For MSC-exosome treatment, PC12 cells were seeded with FBS-free culture medium in 12-well plates and treated with exosomes (100 *μ*g/mL) from transfected and untransfected MSCs at the times indicated.

To mimic ischemic condition *in vitro*, PC12 cells were exposed to OGD for 6 h. PC12 cells were then placed in the original medium with or without PUR (20 *μ*M) and with/without Cyc (10 *μ*M) and returned to the incubator under normoxic conditions for the times indicated.

### 2.3. Determination of Apoptosis by Flow Cytometric Analysis

Apoptosis of PC12 cells was assessed with use of the annexin V-FITC/PI Double Labeling Apoptosis Detection Kit as previously described. The percent of annexin V-positive cells determined over 10,000 acquired events was analyzed with use of a FACS flow cytometer C6 (BD Biosciences, San Jose, CA, USA). All assays were performed in triplicate, and each experiment was repeated three times.

### 2.4. TUNEL Staining

Cellular death was determined with use of TUNEL staining according to the instructions provided and counterstained with DAPI. The number of TUNEL-positive cells was measured in six randomly selected microscopic fields at 200x magnification within the lesion area of each group as described above (*N* = 4 mice/group).

### 2.5. DHE Analysis

For determinations of ROS production in primary neurons, cells were stained with 10 *μ*M DHE for 30 min. After rinsing and mounting, images were captured with the use of fluorescent microscopy (BX51; Olympus, Tokyo, Japan). DHE-staining results were pixilated and quantified with the use of the Image-Pro Plus image analysis system.

### 2.6. Western Blot

Tissues were homogenized with RIPA containing PMSF and protease/phosphatase inhibitors following centrifugation at 4°C at 13,800 × g for 10 min. Then, 5× loading buffer was added to the protein supernatant and total protein was quantified using a BCA assay kit CWBIO (Haimen, Jiangsu, China). Equal amounts of protein were separated by SDS-PAGE and then transferred to PVDF membranes. After blocking in 5% nonfat milk for 2 h, blots were probed using the following primary antibodies: Shh, Gli-1, Patch, p-CREB, CREB, BDNF, p-Akt, Akt, NF-*κ*B, p-NF-*κ*B, and *β*-actin at 4°C overnight. Secondary antibodies were then incubated with the membranes at 37°C for 60 min. The chemiluminescent signal was developed with use of ECL kit reagents (MILLIPORE, USA) and then detected with the use of the Tanon Imaging System (Tanon-4600). Densities of protein bands were semiquantified using ImageJ (National Institutes of Health, Bethesda, MD, USA).

### 2.7. Reverse Transcriptase Quantitative Real-Time PCR (qRT-PCR)

Total RNA of tissue was isolated using TRIzol reagent CWBIO (Haimen, Jiangsu, China) according to the instructions of the manufacturer. Total RNA of EVs and H_2_S-EVs were extracted using the Sera Mir EV RNA Extraction Kit (System Biosciences, USA) after isolation of EVs using ExoQuick-TC™ (System Biosciences, USA). Complementary DNA (cDNA) was synthesized using a reverse transcription system with the ReverTra Ace qPCR RT Kit (TOYOBO, Tokyo, Japan). Quantitative real-time PCR was performed using SYBR green PCR master mix (TOYOBO, Tokyo, Japan) on the Bio-Rad IQ5 Real-Time PCR System (Bio-Rad, USA). The specific primers for BDNF were purchased from RiboBio Co., Ltd. (Guangzhou, China): forward, 5′-AGC TGA GCG TGT GTG ACA GT-3′; reverse, 5′-ACC CAT GGG ATT ACA CTT GG-3′. Results were normalized with U6/actin according to the 2 − ΔΔCt method.

### 2.8. Immunofluorescent Staining

For immunofluorescent staining, cells were incubated with the primary antibody (Gli-1, 1 : 100) overnight at 4°C and with the secondary antibody at 37°C for 30 min on the following day. The nucleus was stained with DAPI for 10 min. Images were obtained with fluorescent microscopy (OLYMPUS-BX51, Olympus Corporation, Japan), and analyses of these images were performed using the Image-Pro Plus 6.0 software (Media Cybernetics, MD, USA).

### 2.9. Animal Model and Treatment

Animal experiments were performed according to the International Guiding Principles for Animal Research provided by the Council for International Organizations of Medical Sciences (CIOMS), and all procedures were approved by the Animal Ethical and Welfare Committee of Shandong University. Participants who worked with the animal models were trained according to procedures within the Institutional Animal Care and Use Committee Guidebook (IACUC). All procedures were executed to minimize the pain experienced by the animals in these protocols.

The middle cerebral artery occlusion (MCAO) procedure was used to generate ischemic injury. Adult male Sprague-Dawley (SD) rats (280-320 g) were randomly divided into four groups (*N* = 14/group): (1) sham, (2) MCAO, (3) MCAO+PUR, and (4) MCAO+PUR+Cyc. Rats were treated with saline, PUR (5 mg/kg), or Cyc (1 mg/kg) and received intraperitoneal injections once per day for 2 days after MCAO. The MACO+PUR+Cyc group were pretreated with Cyc (1 mg/kg) at 30 min before PUR.

MCAO was based on the modified Longa method as previously described [14]. Briefly, for MACO model, rats were anesthetized with isoflurane. The right common carotid artery (CCA), external carotid artery (ECA), and internal carotid artery (ICA) were isolated followed by clamping of the ICA and CCA with microartery clips. The proximal portion of the ECA was ligated using a 5-0 polyester suture and severed at 3.0 mm from the bifurcation of the CCA. The ICA was then completely dissociated, and microsurgical scissors were used to incise a small opening in the arterial wall at 3 mm from the arterial bifurcation at the proximal end of the ECA. A thread embolus was inserted into the ECA parallel with that of the ICA, and the clamp on the ICA was then removed. After achieving microresistance, advancement of the embolus was stopped and the ECA was then tightened with a 5-0 polyester suture. The sham group animals underwent similar surgical procedures without applying the occlusion.

The brain infarct was assessed with use of 2,3,5,-triphenyltetrazolium chloride (TTC) staining at 2 days following injury. Neurological functions and brain water content were assessed at 2 days following injury.

### 2.10. Brain Tissue Water Content Determination

Brain tissues were quickly removed and weighed on an analytical balance with an accuracy of 0.01 mg (wet weight). The hemispheres were then dried in an oven at 105°C for 24 h to obtain the dry weight content [15]. The formula for brain water content was brain water content (%) = [(wet weight − dry weight)/wet weight] × 100%.

### 2.11. Neurobehavioral Tests

Behavior was assessed in a single-blinded manner using the modified Longa method and rated on a scale from 0–4 [14]: 0, no neurological deficit; 1, unable to extend the contralateral forelimb and failure to straighten limb; 2, contralateral forelimb flexion and walking in a circle; 3, leans slightly to the contralateral side and walking in a circle toward the contralateral side; and 4, walking in a circle toward the contralateral side. Animals with scores of 1, 2, or 3 points were selected for the experiment.

### 2.12. TUNEL Analyses

TUNEL analyses of the brain section were determined as described above. Then, the brain sections were performed using the Image-Pro Plus 6.0 software by an investigator blinded as to experimental group assignments. The brain slices in the region containing the infarct lesion (between -1.60 and -2.00 mm from the bregma) were chosen to undergo TUNEL staining. All the slices of each group used in every independent experiment have the similar anatomical positions. The positive cells were counted within randomly selected peri-infarct areas which limited within 300 *μ*m to the infarct.

### 2.13. Statistical Analyses

Results are expressed as mean ± SD. Correlation analysis between the expressions of neuroligin/neurexin and TUNEL counts *in vitro* was performed with Pearson correlation test. Unless otherwise indicated, other data were analyzed using one-way ANOVAs followed by Tukey's test or Dunnett's test for post hoc comparisons using Prism software. A *p* value < 0.05 was required for results to be considered statistically significant.

## 3. Results

### 3.1. OGD Exposure Affects the Shh Signaling Pathway

**Figure 1:**
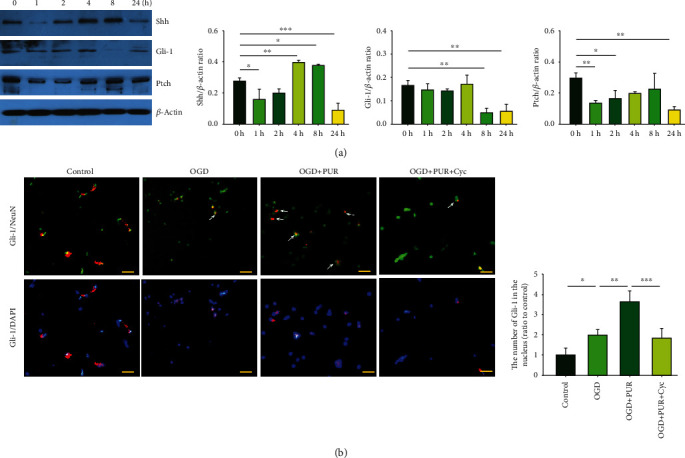
Effects of OGD exposure on the Shh signaling pathway: (a) protein levels of Shh, Gli-1, and patch at 1, 2, 4, 8, and 24 h after OGD exposure as determined by Western blot (*N* = 3/group); (b) nuclear translocation of Gli-1 at 24 h after OGD was observed with the use of immunofluorescent staining (scale bar = 50 *μ*m). Quantification of Gli-1 nuclear translocation (*N* = 4/group). Values represent the mean ± SD; ^∗^*p* < 0.05, ^∗∗^*p* < 0.01, and ^∗∗∗^*p* < 0.001 according to ANOVA according to ANOVA with Dunnett test in (a) and Tukey's post hoc comparisons in (b).

Results from Western blots showed that compared with the control group, OGD exposure produced increased expressions of Shh at 4 h ([*F*(5, 12 = 35.277, *p* < 0.001], post hoc *p* < 0.01) and 8 h (post hoc *p* < 0.05) and decreased expressions at 1 h (post hoc *p* < 0.01) and 24 h (post hoc *p* < 0.001) (Figure 1(a)). Moreover, OGD exposure downregulated the expressions of Gli-1 ([*F*(5, 12 = 12.486, *p* > 0.001], post hoc *p* < 0.01) and Ptch ([*F*(5, 12 = 5.959, *p* > 0.01], post hoc *p* < 0.01) at 24 h. With immunofluorescent staining, PUR treatment was found to promote Gli-1 nuclear translocation ([*F*(3, 12 = 27.524, *p* < 0.001], post hoc *p* < 0.01) (Figure 1(b)). These effects of PUR on the Shh pathway following OGD exposure were blocked by Cyc pretreatment (Figure 1).

### 3.2. Activation of Shh Signals with PUR Attenuates Apoptosis Induced by OGD

**Figure 2:**
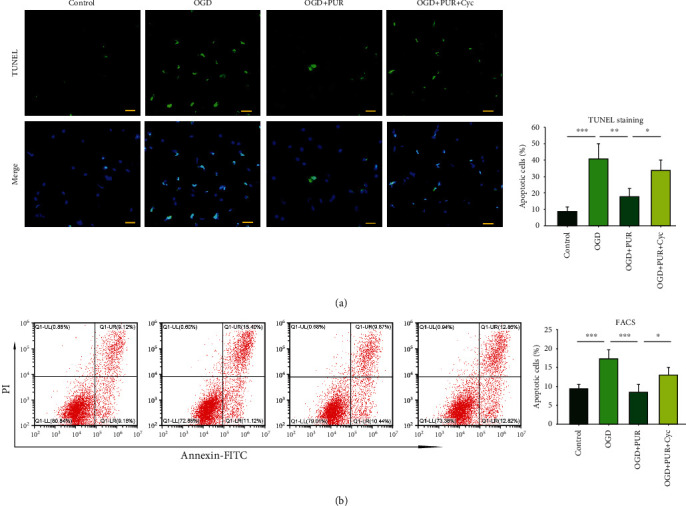
PUR activation of OGD-induced apoptosis: (a) apoptosis as determined by TUNEL staining at 24 h after OGD (*N* = 4/group; scale bar = 50 *μ*m); (b) apoptosis as determined by FACS at 24 h after OGD (*N* = 4/group). Values represent the mean ± SD; ^∗^*p* < 0.05, ^∗∗^*p* < 0.01, and ^∗∗∗^*p* < 0.001 according to ANOVA with Tukey's post hoc comparisons.

Next, we examined whether PUR affected OGD-induced apoptosis in primary neurons using TUNEL staining. OGD significantly increased the percent of apoptotic/total cells (40.6 ± 9.12%) compared with that of the control group (8.6 ± 2.90%) [*F*(3, 12 = 22.202, *p* < 0.001], post hoc *p* < 0.001), while treatment with 20 *μ*M PUR significantly reduced the proportion of TUNEL-positive cells (17.84 ± 4.91%) as compared with the OGD group (post hoc *p* < 0.01). This effect of PUR on the number of TUNEL-positive cells was blocked by Cyc (33.84 ± 6.20%; post hoc *p* < 0.05) (Figure 2(a)).

Results of FACS showed that OGD exposure increased apoptosis in PC12 cells/total (17.4 ± 2.32%) as compared with the control group (9.41 ± 1.09%) [*F*(3, 12 = 18.403, *p* < 0.001], post hoc *p* < 0.001). Treatment with 20 *μ*M PUR significantly reduced the proportion of TUNEL-positive cells (8.46% ± 1.97) as compared with the OGD group (post hoc *p* < 0.001). This effect of PUR on the number of TUNEL-positive cells was blocked by Cyc (13.03 ± 1.97%; post hoc *p* < 0.05) (Figure 2(b)).

### 3.3. Activation of Shh Signals Reduces OGD-Induced ROS Generation

**Figure 3:**
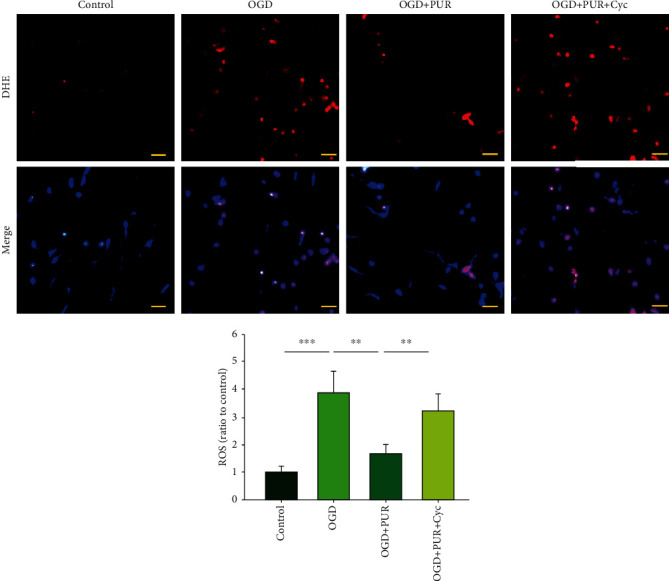
PUR activation of Shh on OGD-induced ROS generation. ROS levels were determined by DHE staining at 24 h after OGD (*N* = 4/group; scale bar = 50 *μ*m). Values represent the mean ± SD; ^∗∗^*p* < 0.01 and ^∗∗∗^*p* < 0.001 according to ANOVA with Tukey's post hoc comparisons.

Compared to the control group, OGD significantly increased ROS levels as determined at 24 h following OGD ([*F*(3, 12 = 7.248, *p* < 0.001], post hoc *p* < 0.001), while PUR treatment significantly attenuated this OGD-induced increase in ROS levels (post hoc *p* < 0.01). This effect of PUR on ROS generation was blocked by Cyc (post hoc *p* < 0.01) (Figure 3).

### 3.4. Activation of Shh Signals with PUR Attenuates OGD-Induced Reductions in Neuroligin and Neurexin

**Figure 4:**
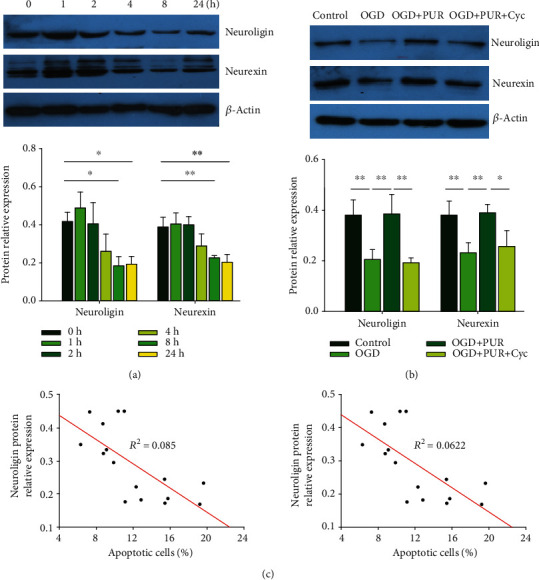
PUR activation of Shh on OGD-induced neuroligin and neurexin: (a) protein levels of neuroligin and neurexin at 1, 2, 4, 8, and 24 h after OGD as determined by Western blot (*N* = 3/group); (b) protein levels of neuroligin and neurexin at 24 h after OGD as determined by Western blot (*N* = 4/group); (c) Pearson correlation coefficients obtained between neuroligin/neurexin expressions and apoptosis following PUR treatment. Values represent the mean ± SD; ^∗^*p* < 0.05 and ^∗∗^*p* < 0.01 according to ANOVA with Dunnett test in (a) and Tukey's post hoc comparisons in (b).

As synaptic proteins (neuroligin and neurexin) play a crucial role in stimulating synapse formation and reconstruction [16], we next examined the effects of OGD on the expressions of neuroligin and neurexin. Results from the Western blot assay demonstrated that neurons exposed to OGD showed a significant decrease in neuroligin expression at 8 h ([*F*(5, 12 = 8.841, *p* < 0.01], post hoc *p* < 0.05) and at 24 h (post hoc *p* < 0.05) post-OGD. Neurexin expression was also decreased at 8 h ([*F*(5, 12 = 10.839, *p* < 0.001], post hoc *p* < 0.01) and at 24 h (post hoc *p* < 0.01) after OGD exposure (Figure 4(a)). PUR treatment upregulated these OGD-induced reductions in neuroligin ([*F*(3, 12 = 15.590, *p* < 0.001], post hoc *p* < 0.01) and neurexin ([*F*(3, 12 = 11.268, *p* < 0.01], post hoc *p* < 0.01) at 8 h after OGD (Figure 4(b)). These effects of PUR on neuroligin and neurexin expression were blocked by Cyc (neuroligin, post hoc *p* < 0.01; neurexin, post hoc *p* < 0.05).

Moreover, increasing expressions of neuroligin and neurexin were found to be negatively correlated with attenuating levels of apoptosis following PUR treatment (neuroligin, Pearson *r* = 0.686, *p* = 0.003; neurexin, Pearson *r* = 0.684, *p* = 0.004) (Figure 4(c)).

### 3.5. Activation of Shh Signals Increases CREB and BDNF Expressions after OGD

**Figure 5:**
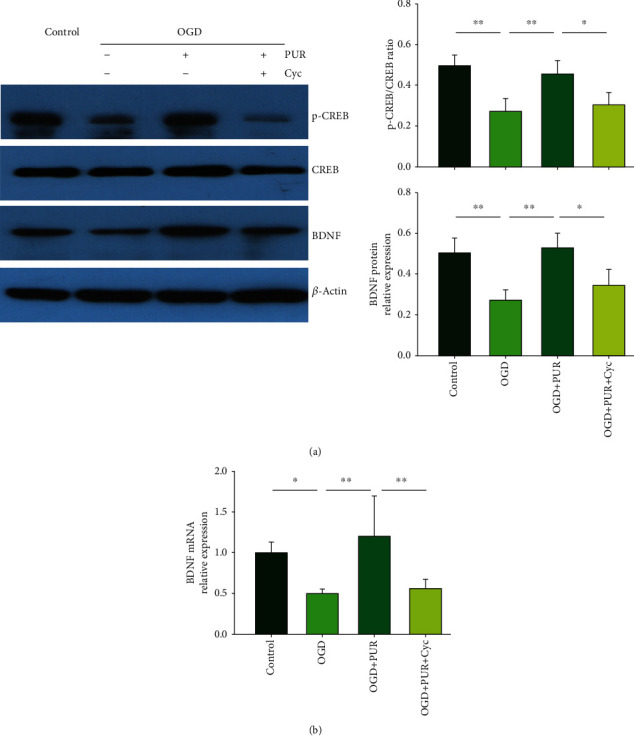
PUR activation of Shh on OGD-induced CREB and BDNF expressions: (a) protein levels of p-CREB, CREB, and BDNF at 4 h after OGD as determined with the use of Western blot (*N* = 3/group); (b) the level of BDNF mRNA at 4 h after OGD as determined with the use of qRT-PCR (*N* = 3/group). Values represent the mean ± SD, ^∗^*p* < 0.05, and ^∗∗^*p* < 0.01 according to ANOVA with Tukey's post hoc comparisons.

Compared to the control group, OGD significantly decreased p-CREB expression at 4 h ([*F*(3, 12 = 12.837, *p* < 0.001], post hoc *p* < 0.01) and BDNF expression at 24 h ([*F*(3, 12 = 13.288, *p* < 0.001], post hoc *p* < 0.01) following OGD exposure. However, in response to PUR treatment, both p-CREB (post hoc *p* < 0.01) and BDNF (post hoc *p* < 0.01) expressions were significantly increased (Figure 5(a)). These effects of PUR on p-CREB and BDNF were blocked by Cyc (p-CREB, post hoc *p* < 0.05; BDNF, post hoc *p* < 0.05).

BDNF mRNA was also significantly decreased at 4 h after OGD exposure ([*F*(3, 12 = 9.856, *p* < 0.001], post hoc *p* < 0.05), while treatment with PUR increased this BDNF mRNA expression (post hoc *p* < 0.01) (Figure 5(b)).

### 3.6. Activation of Shh Signals with PUR Promotes p-Akt and Reduces p-NF-*κ*B

**Figure 6:**
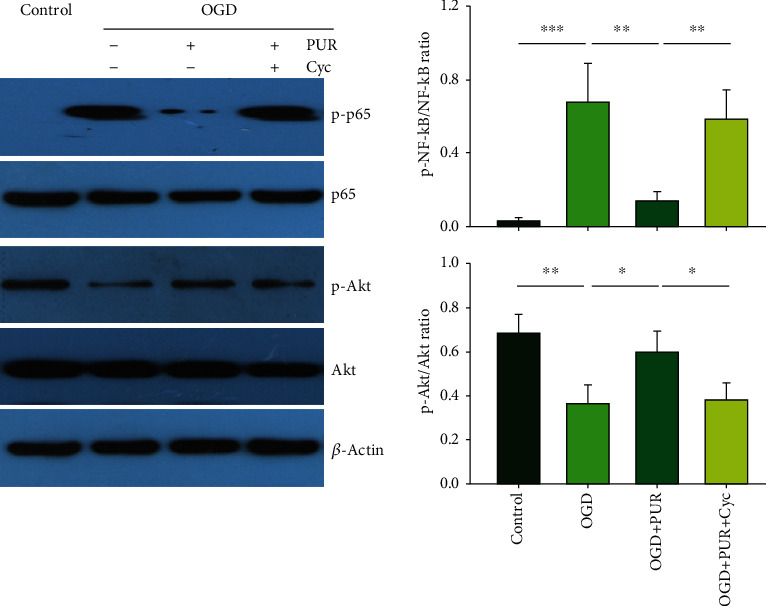
PUR activation of Shh on OGD-induced p-Akt and p-NF-*κ*B. Protein levels of p-Akt, Akt, p-NF-*κ*B, and NF-*κ*B at 4 h after OGD as determined with use of Western blot (*N* = 4/group). Values represent the mean ± SD; ^∗^*p* < 0.05, ^∗∗^*p* < 0.01, and ^∗∗∗^*p* < 0.001 according to ANOVA with Tukey's post hoc comparisons.

Compared with the control group, the OGD exposure group showed a significantly decreased expression of p-Akt ([*F*(3, 12 = 13.045, *p* < 0.001], post hoc *p* < 0.01) and significantly increased expression of p-NF-*κ*B ([*F*(3, 12 = 22.439, *p* < 0.001], post hoc *p* < 0.001) at 4 h post-OGD. PUR treatment increased both p-Akt (post hoc *p* < 0.05) and p-NF-*κ*B (post hoc *p* < 0.01) expressions (Figure 6). These effects of PUR on p-Akt and p-NF-*κ*B were blocked by Cyc (p-Akt, post hoc *p* < 0.05; p-NF-*κ*B, post hoc *p* < 0.01) (Figure 6).

### 3.7. PUR Treatment Alleviates Neurologic Deficits, Edema, and Apoptosis after Ischemic Injury

**Figure 7:**
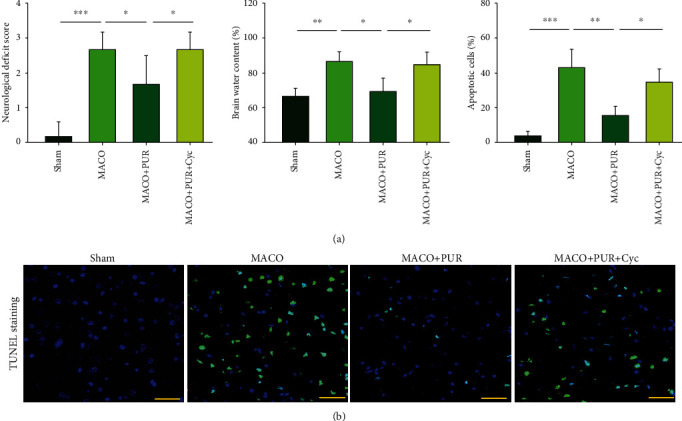
PUR activation of Shh on neurological recovery after MCAO. (a) Neurological function within each group was assessed at 48 h after MCAO (*N* = 6/group). Brain water content in rats from each group at 48 h (*N* = 4/group). (b) Apoptosis was determined with the use of TUNEL staining at 48 h after MACO injury (*N* = 4/group; scale bar = 50 *μ*m). Values represent the mean ± SD, ^∗^*p* < 0.05, ^∗∗^*p* < 0.01, and ^∗∗∗^*p* < 0.001 according to ANOVA with Tukey's post hoc comparisons.

Neurological deficit scores of the MCAO group were significantly lower than those of the sham group at 48 h ([*F*(3, 20 = 24.512, *p* < 0.001], post hoc *p* < 0.01), while PUR improved these deficits within the MCAO group at 24 h and 48 h after treatment (post hoc *p* < 0.01) (Figure 7(a)). Ischemic insult significantly increased the water content within the ipsilateral side of the brain ([*F*(3, 12 = 10.797, *p* < 0.01], post hoc *p* < 0.01), and PUR treatment markedly reduced this ischemic-induced brain edema as compared with that observed in the MACO group (post hoc *p* < 0.05) (Figure 7(b)).

In addition, this ischemic insult significantly increased the number of apoptotic cells within the ipsilateral side ([*F*(3, 12 = 26.826, *p* < 0.001], post hoc *p* < 0.001), an effect which was significantly reduced in response to PUR treatment (post hoc *p* < 0.01) (Figure 7(c)). These effects of PUR on neurological deficit scores, edema, and apoptosis were all blocked by Cyc (post hoc *p* < 0.05, for each) (Figure 7).

## 4. Discussion

In the present study, we demonstrate that activation of Shh signals was beneficial for neurological recovery after MCAO in rats. Moreover, we show that these beneficial effects of Shh signaling in response to ischemic exposure were associated with enhanced neuronal viability, increased neuroligin and neurexin expression along with activated Akt, and decreased NF-*κ*B signaling.

Overall, alterations in Shh signaling have been shown to be related with a number of regenerative responses and postinjury pathophysiologies after trauma within diverse regions of the CNS [17]. For example, the Shh pathway is maximally activated at 72 h in response to brain injury followed by a return to baseline levels at 14 days [18] and cortical Shh protein levels are increased at 1 to 5 days after a cortical stab wound injury [19]. There is one report showing that expressions of Shh, Gli-1, and Ptch1 protein were all upregulated in cortical neurons at 6 h after MACO injury in rats [10], while others have reported that Gli1 and Ptch1 expressions were upregulated at 6, 12, 24, and 48 h postischemic injury [20]. However, a downregulation of Shh expression within the cortex has also been reported in the early stages after experimental subarachnoid hemorrhage [11, 21] and hypoxia-ischemia in neonatal mice [12]. In the present study, we found that neuronal expressions of Shh and Gli-1 were increased in the early stages of OGD insult, while the expressions of Shh, Gli-1, and Ptch were decreased at later time points following OGD. Activation of the Shh signaling pathway has been shown to increase Bcl-2, while suppressing Bax expression [22]. Moreover, exogenous Shh treatment reduces infarct volume along with promoting angiogenesis and neuronal survival after MACO injury [23]. Our current results demonstrate that restoring expression of the Shh signaling pathway with PUR played a protective role against neurotoxicity after ischemic exposure. That this neuroprotective effect did involve a PUR-induced activation of the Shh signaling pathway was substantiated from results obtained with Cyc treatment, which reversed these beneficial effects of PUR upon ischemic exposure. Thus, we concluded that the upregulation of Shh signaling serves to resist ischemic injury in the early stages thereby exerting its beneficial therapeutic effects in cerebral ischemic stroke, while it is then decreased in the later stages due to the aggravation resulting from the injury.

Results from a previous study have demonstrated that PUR exhibits beneficial effects against stroke insult in rodent models and PUR does not alter the stroke-induced level of Shh signaling [10]. PUR plays an antiapoptotic role in the early stage by targeting neurons and also plays an anti-inflammatory role in late-stage inflammation by targeting astrocytes [10]. In addition, we found that PUR can reverse the expression of Shh signaling following OGD exposure in neurons *in vitro*. One explanation for this discrepancy likely comes from the different cell targets of PUR in the CNS following ischemic injury.

The CNS exhibits a substantial degree of plasticity after injury which then enables it to recover from functional deficits via processes involving neurogenesis, angiogenesis, axonal sprouting, and synaptic formation and remodeling. Activation of Shh receptors in the dendrites of hippocampal neurons has been shown to accelerate axonal elongation and synaptic plasticity after ischemic stroke [24]. The Shh pathway can mediate brain plasticity and functional recovery via plasminogen activator which, in part, explains the functional recovery observed after treatment of stroke with bone marrow stromal cells [25]. Moreover, exogenous Shh treatment increases the levels of BDNF and promotes nerve regeneration after cavernous nerve injury [26]. In line with these findings, our present results showed that activation of Shh signaling with PUR increased p-CREB and BDNF expression in response to OGD exposure, effects which were associated with an upregulation in the synaptic proteins, neuroligin and neurexin. The presynaptic neurexin and postsynaptic neuroligin are synaptic cell-adhesion molecules that connect neurons at synapses and regulate synaptic transmission [27]. Ischemia injury has been reported to increase neurexin-neuroligin1-PSD-95 interactions, which may represent an important target for therapeutic agents directed at the treatment of brain ischemia [28]. In the present study, restoring the expression of Shh signaling with PUR upregulated the expressions of neuroligin and neurexin, along with promoting cell survival and modulation of CREB/BDNF pathways. These data suggest that Shh signaling was able to mitigate ischemic injury via affects upon neurotrophic responses and synaptic plasticity.

Phosphorylated Akt is closely related to diverse cellular functions, such as cellular survival, apoptosis, and metabolism. The hedgehog signaling pathway component of Gli-1 can activate the PI3K-Akt pathway [29]. In line with this finding, we demonstrated that PUR activated Akt following OGD exposure in neurons. NF-*κ*B induces the expressions of inflammation cytokines in astrocytes via the Shh signaling pathway [30]. The Shh ligand in the bone marrow microenvironment was involved in promoting NF-*κ*B activity in multiple myeloma cells [31]. We found that PUR decreased OGD-induced phosphorylated NF-*κ*B, associated with antiapoptotic effects. We speculated that the Akt and NF-*κ*B pathways were responsible for PUR's neuroprotective activity, which needs further study.

Collectively, our data indicate that the Shh signaling pathway plays a significant neuroprotective role against ischemic injury by promoting synaptic and neuronal health.

## Data Availability

The raw/processed data required to reproduce these findings cannot be shared at this time as the data also forms part of an ongoing study.

## Abbreviations

CNS: Central nervous system
CCA: Right common carotid artery
Cyc: Cyclopamine
ECA: External carotid artery
Gli-1: Glioma-associated oncogene homolog 1
ICA: Internal carotid artery
MCAO: Middle cerebral artery occlusion
OGD: Oxygen-glucose deprivation
PUR: Purmorphamine
Ptch: Patched
qRT-PCR: Reverse transcriptase quantitative real-time PCR
Smo: Smoothened
Shh: Sonic hedgehog
TUNEL: Terminal deoxynucleotidyl transferase-mediated dUTP-biotin nick end labeling.

## References

[1] Moretti A., Ferrari F., Villa R. F. (2015). Neuroprotection for ischaemic stroke: current status and challenges. *Pharmacology & Therapeutics*.

[2] Rodrigo R., Fernandez-Gajardo R., Gutierrez R. (2013). Oxidative stress and pathophysiology of ischemic stroke: novel therapeutic opportunities. *CNS & Neurological Disorders Drug Targets*.

[3] Wilson N. H., Stoeckli E. T. (2012). Sonic hedgehog regulates Wnt activity during neural circuit formation. *Vitamins and Hormones*.

[4] Ho K. S., Scott M. P. (2002). Sonic hedgehog in the nervous system: functions, modifications and mechanisms. *Current Opinion in Neurobiology*.

[5] Liu L., Zhao B., Xiong X., Xia Z. (2018). The neuroprotective roles of sonic hedgehog signaling pathway in ischemic stroke. *Neurochemical Research*.

[6] Sims J. R., Lee S. W., Topalkara K. (2009). Sonic hedgehog regulates ischemia/hypoxia-induced neural progenitor proliferation. *Stroke*.

[7] Ji H., Miao J., Zhang X. (2012). Inhibition of sonic hedgehog signaling aggravates brain damage associated with the down-regulation of Gli 1, Ptch 1 and SOD1 expression in acute ischemic stroke. *Neuroscience Letters*.

[8] Sinha S., Chen J. K. (2006). Purmorphamine activates the Hedgehog pathway by targeting Smoothened. *Nature Chemical Biology*.

[9] Alvarez J. I., Dodelet-Devillers A., Kebir H. (2011). The Hedgehog pathway promotes blood-brain barrier integrity and CNS immune quiescence. *Science*.

[10] Chechneva O. V., Mayrhofer F., Daugherty D. J. (2014). A Smoothened receptor agonist is neuroprotective and promotes regeneration after ischemic brain injury. *Cell Death & Disease*.

[11] Hu Q., Li T., Wang L. (2017). Neuroprotective effects of a Smoothened receptor agonist against early brain injury after experimental subarachnoid hemorrhage in rats. *Frontiers in Cellular Neuroscience*.

[12] Liu D., Bai X., Ma W. (2020). Purmorphamine attenuates neuro-inflammation and synaptic impairments after hypoxic-ischemic injury in neonatal mice via Shh signaling. *Frontiers in Pharmacology*.

[13] Wang Z., Liu D., Wang F. (2012). Saturated fatty acids activate microglia via Toll-like receptor 4/NF-*κ*B signalling. *The British Journal of Nutrition*.

[14] Longa E. Z., Weinstein P. R., Carlson S., Cummins R. (1989). Reversible middle cerebral artery occlusion without craniectomy in rats. *Stroke*.

[15] Shao A., Guo S., Tu S. (2014). Astragaloside IV alleviates early brain injury following experimental subarachnoid hemorrhage in rats. *International Journal of Medical Sciences*.

[16] Elegheert J., Cvetkovska V., Clayton A. J. (2017). Structural mechanism for modulation of synaptic neuroligin-neurexin signaling by MDGA proteins. *Neuron*.

[17] Mierzwa A. J., Sullivan G. M., Beer L. A., Ahn S., Armstrong R. C. (2014). Comparison of cortical and white matter traumatic brain injury models reveals differential effects in the subventricular zone and divergent sonic hedgehog signaling pathways in neuroblasts and oligodendrocyte progenitors. *ASN Neuro*.

[18] Amankulor N. M., Hambardzumyan D., Pyonteck S. M., Becher O. J., Joyce J. A., Holland E. C. (2009). Sonic hedgehog pathway activation is induced by acute brain injury and regulated by injury-related inflammation. *The Journal of Neuroscience: The Official Journal of the Society for Neuroscience*.

[19] Sirko S., Behrendt G., Johansson P. A. (2013). Reactive glia in the injured brain acquire stem cell properties in response to sonic hedgehog. *Cell Stem Cell*.

[20] Ji H., Zhang X., Du Y., Liu H., Li S., Li L. (2012). Polydatin modulates inflammation by decreasing NF-*κ*B activation and oxidative stress by increasing Gli1, Ptch1, SOD1 expression and ameliorates blood-brain barrier permeability for its neuroprotective effect in pMCAO rat brain. *Brain Research Bulletin*.

[21] Zuo S., Li W., Li Q. (2015). Protective effects of Ephedra sinica extract on blood-brain barrier integrity and neurological function correlate with complement C3 reduction after subarachnoid hemorrhage in rats. *Neuroscience Letters*.

[22] Dai R. L., Zhu S. Y., Xia Y. P. (2011). Sonic hedgehog protects cortical neurons against oxidative stress. *Neurochemical Research*.

[23] Chen S. C., Huang M., He Q. W. (2017). Administration of sonic hedgehog protein induces angiogenesis and has therapeutic effects after stroke in rats. *Neuroscience*.

[24] Yao P. J., Petralia R. S., Mattson M. P. (2016). Sonic hedgehog signaling and hippocampal neuroplasticity. *Trends in Neurosciences*.

[25] Ding X., Li Y., Liu Z. (2013). The sonic hedgehog pathway mediates brain plasticity and subsequent functional recovery after bone marrow stromal cell treatment of stroke in mice. *Journal of Cerebral Blood Flow and Metabolism*.

[26] Bond C. W., Angeloni N., Harrington D., Stupp S., Podlasek C. A. (2013). Sonic hedgehog regulates brain-derived neurotrophic factor in normal and regenerating cavernous nerves. *The Journal of Sexual Medicine*.

[27] Sudhof T. C. (2008). Neuroligins and neurexins link synaptic function to cognitive disease. *Nature*.

[28] Li C., Han D., Zhang F., Zhou C., Yu H. M., Zhang G. Y. (2007). Preconditioning ischemia attenuates increased neurexin–neuroligin1–PSD-95 interaction after transient cerebral ischemia in rat hippocampus. *Neuroscience Letters*.

[29] Kim C. S., Vasko V. V., Kato Y. (2005). AKT activation promotes metastasis in a mouse model of follicular thyroid carcinoma. *Endocrinology*.

[30] Chen K. Y., Wang L. C. (2017). Stimulation of IL-1*β* and IL-6 through NF-*κ*B and sonic hedgehog-dependent pathways in mouse astrocytes by excretory/secretory products of fifth-stage larval Angiostrongylus cantonensis. *Parasites & Vectors*.

[31] Cai K., Na W., Guo M. (2019). Targeting the cross-talk between the hedgehog and NF-*κ*B signaling pathways in multiple myeloma. *Leukemia & Lymphoma*.

