# Corrigendum to “Neuroprotective Effects of the Sonic Hedgehog Signaling Pathway in Ischemic Injury through Promotion of Synaptic and Neuronal Health”

**DOI:** 10.1155/2021/9762592

**Published:** 2021-05-25

**Authors:** Sen Yin, Xuemei Bai, Danqing Xin, Tingting Li, Xili Chu, Hongfei Ke, Min Han, Wenqiang Chen, Xingang Li, Zhen Wang

**Affiliations:** ^1^Qilu Hospital, Cheeloo College of Medicine, Shandong University, Jinan, Shandong, China; ^2^Department of Physiology, School of Basic Medical Sciences, Cheeloo College of Medicine, Shandong University, Jinan, Shandong 250012, China

In the article titled “Neuroprotective Effects of the Sonic Hedgehog Signaling Pathway in Ischemic Injury through Promotion of Synaptic and Neuronal Health” [1], the authors have identified that in Figure 4, the incorrect images were provided due to an error during the preparation of the manuscript. The authors confirm that this error does not affect the results of the article and the corrected Figure 4 is shown below.

## Figures and Tables

**Figure 1:**
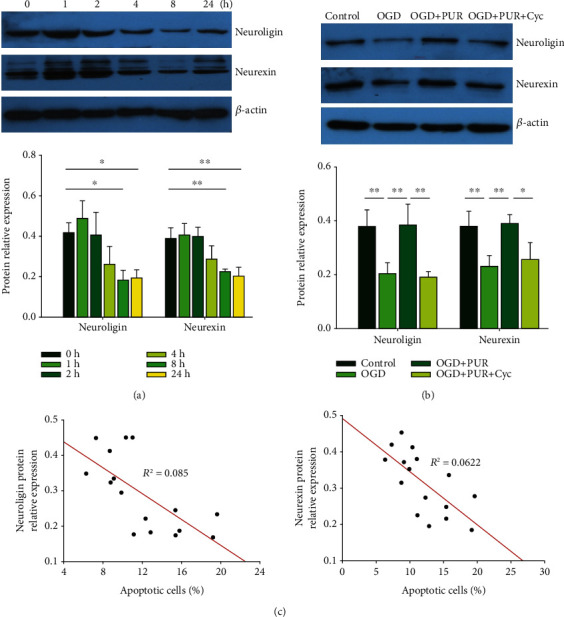
PUR activation of Shh on OGD-induced neuroligin and neurexin: (a) protein levels of neuroligin and neurexin at 1, 2, 4, 8, and 24 h after OGD as determined by Western blot (*N* = 3/group); (b) protein levels of neuroligin and neurexin at 24 h after OGD as determined by Western blot (*N* = 4/group); (c) Pearson correlation coefficients obtained between neuroligin/neurexin expressions and apoptosis following PUR treatment. Values represent the mean ± SD; ^∗^*p* < 0.05 and ^∗∗^*p* < 0.01 according to ANOVA with the Dunnett test in (a) and Tukey's post hoc comparisons in (b).
